# Prevalence and sociodemographic associations of common mental disorders in a nationally representative sample of the general population of Greece

**DOI:** 10.1186/1471-244X-13-163

**Published:** 2013-06-04

**Authors:** Petros Skapinakis, Stefanos Bellos, Sotirios Koupidis, Ilias Grammatikopoulos, Pavlos N Theodorakis, Venetsanos Mavreas

**Affiliations:** 1 Department of Psychiatry, University of Ioannina, School of Medicine, Ioannina 45110, Greece

## Abstract

**Background:**

No study in Greece has assessed so far the full range of common mental disorders using a representative sample of the population from both mainland and insular regions of the country. The aim of the present paper was to present the results of the first such study.

**Methods:**

The study was carried out between 2009–2010 in a nationally representative sample of 4894 individuals living in private households in Greece. Common mental disorders in the past week were assessed with the revised Clinical Interview Schedule (CIS-R). We also assessed alcohol use disorders (using AUDIT), smoking and cannabis use.

**Results:**

14% of the population (Male: 11%, Female: 17%) was found to have clinically significant psychiatric morbidity according to the scores on the CIS-R. The prevalence (past seven days) of specific common mental disorders was as follows: Generalized Anxiety Disorder: 4.10% (95% CI: 3.54, 4.65); Depression: 2.90% (2.43, 3.37); Panic Disorder: 1.88% (1.50, 2.26); Obsessive-Compulsive Disorder: 1.69% (1.33, 2.05); All Phobias: 2.79% (2.33, 3.26); Mixed anxiety-depression: 2.67% (2.22, 3.12). Harmful alcohol use was reported by 12.69% of the population (11.75, 13.62). Regular smoking was reported by 39.60% of the population (38.22, 40.97) while cannabis use (at least once during the past month) by 2.06% (1.66, 2.46). Clinically significant psychiatric morbidity was positively associated with the following variables: female gender, divorced or widowed family status, low educational status and unemployment. Use of all substances was more common in men compared to women. Common mental disorders were often comorbid, undertreated, and associated with a lower quality of life.

**Conclusions:**

The findings of the present study can help in the better planning and development of mental health services in Greece, especially in a time of mental health budget restrictions.

## Background

During the past 30 years several epidemiological surveys around the world have shown that mental disorders are a major source of disability accounting for approximately 12% of the global burden of disease [1]. Among these disorders, depression and anxiety disorders (often referred to as “common mental disorders”) are perhaps the most important from a public health perspective due to their prevalence and persistence even in unselected samples of the general population [1,2]. According to projections in 2030 depression will be the leading cause of disability globally accounting for approximately 6% of the total disability [3]. Regarding anxiety disorders, two of the most disabling disorders, panic disorder and obsessive compulsive disorder are associated with almost the same amount of disability as schizophrenia. Alcohol use disorders are also in the list of the top twenty leading causes of disability worldwide [1,3].

Given these figures it is important that national health systems are better organized for the optimal management of the common mental disorders or harmful substance use. Surveys of the general population are the first step in this process and are valuable in providing unbiased and representative information on the prevalence and associations of mental disorders in a defined geographical area [4]. Several countries in Europe and elsewhere in the world have already conducted large epidemiological surveys for these reasons [5,6].

In Greece, no study so far has assessed the full range of common mental disorders using a representative sample of the population from both mainland and insular regions of the country. Two previous nationwide studies were conducted in 1978 and 1984 [7] but these focused mainly in the assessment of general psychiatric morbidity, they used an instrument that predated modern diagnostic criteria (Langner’s scale) and, regarding specific diagnoses, they only covered the diagnosis of depression. In addition, only households from mainland Greece were sampled. More recently, a series of telephone surveys were conducted, but these also focused on depression only [8,9]. Therefore, there is a need for a large scale, population-based, survey of psychiatric disorders in the whole country, using a detailed diagnostic interview to assess the prevalence and associations of common mental disorders and harmful substance use.

The present study was designed to fulfill this aim. The current paper reports the prevalence, comorbidity, use of services and basic sociodemographic associations of the common mental disorders in Greece. We also report basic data on smoking, harmful alcohol use and cannabis use. A companion paper will focus more specifically in the association between socioeconomic status, subjective financial difficulties, unemployment and common mental disorders (Skapinakis et al., in preparation).

## Methods

### General description of the study

The cross-sectional study reported here is part of the 2009–2010 Psychiatric Morbidity Survey carried out in Greece using a nationally representative sample of the adult population (18 – 70 years). The study was organized by the Ministry of Health and carried out by the University of Ioannina. Data collection was conducted between September 2009 and February 2010. Regarding provision of healthcare in Greece, there are 7 “Regional Health Authorities” (RHA) covering all geographic regions of the country. Eligible for participation were all adults living in households in each of the seven RHAs. Due to the high costs incurred by sampling all islands of the Aegean, Crete was excluded from the sampling (as Crete has been covered in the past by other smaller scale surveys), therefore the sample included participants from all areas of Greece excluding Crete.

### Sampling procedure

Sampling methodology was designed and implemented by a research agency in Greece with substantial experience in conducting nationwide surveys of social or political issues using representative samples of the general adult population of Greece. According to the latest Population Census (2001) the survey population consisted of approximately 7,200,000 individuals. A three-stage sampling design was used with enumerator areas (one or more unified city blocks) based on the 2001 census survey selected at the first stage, households within the selected areas at the second stage and individuals within the households at the third stage.

The primary sampling units (enumerator areas) were first stratified by allocating the Municipalities and Communes included in each Region according to the degree of urbanization (stratum 1: urban areas; stratum 2: semi-urban; stratum 3: rural areas). The stratification for the two major cities, Athens and Thessaloniki, were different (Athens was divided into 31 strata of equal size and Thessaloniki into 9 strata). The projected sample size for the whole survey was 9,800 individuals with a sampling fraction 1/λ for each stratum considered constant and equal to 0.085%.

At the first stage of the sampling procedure primary sampling units (enumerator areas) had a probability of being selected proportional to their size (number of households according to the 2001 census). At the second stage from each selected area (primary sampling unit) the sample of secondary units (households) was selected. Actually, in the second stage a random systematic sample of households was drawn. Systematic sampling is functionally similar to random sampling because each element (household) had a known and equal probability to be selected. Systematic sampling starts by selecting a random starting point (using maps of the enumerator areas) and then every kth element in the sampling frame is selected, where k is the sampling interval. In any selected primary unit, the sample size was determined from the sampling interval which was calculated using data from the 1st stage. At the third stage one eligible member (aged 18–70 years) of the household was selected using simple random sampling.

### Data collection and response rate

In each RHA a pool of 20 trained researchers and 2 supervisors were employed. All instruments used were computerized and responses to the questions/interview were entered directly to a laptop computer. The average time for completion of the instrument was from 30 to 45 minutes depending on the psychopathology. Approximately 35% of the participants entered their data into the laptop without any further assistance from the interviewer after the first guidance. The remaining 65% required some help from the interviewer.

Overall response rate was 54% with a range between 51% and 60% between regions. Refusals were more common from women and the middle aged participants (40–55). Differences between the sample and the 2001 census population data were small. A full detail of the study design, sampling procedures, sampling distribution within each regional health authority and data collection are available from the technical reports submitted to the Ministry of Health and are available by the authors on request.

### Measurement of psychiatric morbidity

Psychiatric morbidity was assessed with the revised clinical interview schedule (CIS-R), a fully structured psychiatric interview designed to be used by trained lay interviewers [10]. The CIS-R was the main instrument used in the national psychiatric morbidity surveys in the UK [11] and has been used in several other similar surveys around the world [12-15]. A computerized version has also been developed and found to be comparable with the regular interview [16]. The CIS-R assesses the presence and severity of 14 different common psychiatric symptoms during the past 7 days (psychosomatic symptoms, fatigue, concentration/memory problems, sleep problems, irritability, worry about physical health, depressive mood, depressive ideas, general worry, free-floating anxiety, phobias, panic, compulsions and obsessions). Two screening questions in each section ask about the presence of the symptom during the past month and then there is a more detailed assessment of the presence, frequency, duration, and severity of the symptom during the past seven days. Additional questions, including questions assessing the impairment of functioning, enable the diagnosis of six common mental disorders (depressive episode, generalized anxiety disorder, all phobias combined, panic disorder, obsessive compulsive disorder, mixed anxiety and depression disorder) according to the ICD-10 research diagnostic criteria using specially developed computerized algorithms.

The Greek version of the CIS-R has been validated and its psychometric properties have been published elsewhere [17]. Each symptom section is scored from 0 to 4 (except depressive ideas from 0 to 5) and a score of 2 or more denotes a clinically significant symptom [10]. Using the CIS-R psychiatric morbidity can be assessed either in a dimensional way, using the total score on the CIS-R (by adding-up all 14 symptom dimensions), or in a categorical form using the six diagnostic categories. For the purposes of the present study we have selected to use both in our analyses in order to be able to investigate potential differences between general psychopathology and specific diagnostic categories. For the dimensional assessment, we have defined four groups of severity based on previous work with the UK and Greek samples [10,11,17]: “no/minimal symptoms” (CIS-R score = 0-5), “subthreshold symptoms” (CIS-R score = 6-11), “mild symptoms” (CIS-R score = 12-17) and “severe symptoms” (CIS-R score > =18). A score on the CIS-R ≥12 (by combining the last two groups into one) is usually considered as the cut-off for “clinically significant” psychiatric morbidity [10,11,17].

### Assessment of substance use

Alcohol- related disorders were assessed with AUDIT [18]. In the present study we used the first three questions in AUDIT (consumption, frequency, binge drinking) to calculate the AUDIT-C subscale with a range of scores from 0 to 12. The AUDIT-C is considered a reliable alcohol screen for use in general population surveys to identify people with hazardous drinking or active alcohol abuse and dependence [19]. To define harmful alcohol use we used the cut points suggested by Aalto et al. [20] who used data from a general population survey. These were a score of ≥ 6 for men and ≥ 4 for women. The cut points are different for the two genders as this has been supported by recent research findings on AUDIT [21].

Current smoking status and current (past-month) cannabis use was obtained from the participants by direct questioning. Regarding smoking, participants were asked to report the average number of cigarettes they smoked per day during the past month. A second question asked the participants to classify themselves into one of the following categories: never-smoker, ex-smoker, occasional/light smoker, moderate smoker, heavy smoker. We combined those two questions to define a binary variable of “regular smoker” in the past 30 days (all those who were at least moderate smokers OR reported more than 2 cigarettes per day on average during the past month). Regarding cannabis, we asked two questions, the first for lifetime use (five categories: never, 1–2 times, 3–10 times, >10 times/regular use, do not wish to reply) and the second for past 30 days use (“have you used cannabis during the past 30 days?” with three possible answers: Yes, No, do not wish to reply). We classified participants as users of cannabis during the past 30 days if they replied yes to the second question OR reported regular use to the first.

### Assessment of health status

We assessed current health status with the EuroQoL EQ-5D, a generic, preference-based, measure of health-related quality of life [22]. This has been validated in Greece by Kontodimopoulos et al. [23]. For the purposes of the present paper we calculated the EQ-5D index scores based on responses to the 5-item questionnaire. The scoring algorithm for the EQ-5D index descriptive system used in this paper is based on UK community preferences as analogous preferences are lacking in Greece [23]. The mean (SD) value of the EQ index in the present sample was 0.82 (0.23) and was very similar to the value of 0.80 (0.27) reported by Kontodimopoulos et al. [23] in their validation study.

### Assessment of course and mental health service use

Persistence of illness was assessed by asking the participants to retrospectively assess the duration of their symptoms. Use of mental health services was assessed by asking participants whether they had visited a mental health professional (either a psychiatrist or psychologist) during the past 12 months for any reason concerning their mental health.

### Assessment of socio-demographic and other variables

Information about sex, age, marital status, employment status and educational qualifications were obtained from the participants. Regarding employment status, we distinguished between unemployment (i.e. the participant did not do any paid work but looked for any kind of paid work in the past 4 weeks) and economic inactivity (the participant did not do any paid work but did not look for any paid work in the past 4 weeks; additional questions clarified the reason for not seeking any work: a) looking after the house, b) retired, and c) a residual category of “other economically inactive” (including students, persons doing their mandatory military service, those living with parents, those unable to work, living on other income such as rents or shares and other non-specific reasons). Participants were also presented with a list of chronic and severe medical conditions (cardiovascular diseases including coronary heart disease and stroke, chronic lung diseases, diabetes, any malignancy, chronic kidney disease) and asked to report whether they suffered from them.

### Statistical analysis

Data were weighted to account for the complex sampling design and non-response. We used the survey commands in Stata version 10.0 to calculate prevalence estimates and 95% confidence intervals [24]. These commands take into account the complex sampling design and compute robust standard errors. Associations between the common mental disorders and sociodemographic associations were examined using odds ratios. These and their 95% confidence intervals were calculated with a series of adjusted logistic regression models using the survey commands in Stata 10.0. All evaluations of statistical significance are based on two-sided tests using the 5% level of significance.

## Results

### Description of the sample

Overall 4,894 adults were interviewed (overall response rate 54.2%, see methods for details). Women represented 50.4% of the final sample. Mean age was 42 years with a standard deviation of 15. The majority of the participants were married (61.2%), employed (59.6%), had graduated from senior high school (48.0%) and were living in an urban environment (54.8%). A detailed table of the distribution of the various sociodemographic variables in the whole sample is given in the appendix (Additional file 1: Table S1).

### Prevalence of psychiatric symptoms

**Figure 1:**
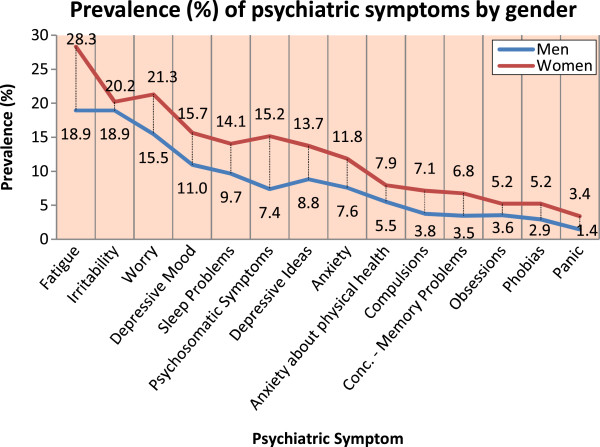
Prevalence of psychiatric symptoms, N = 4894.

**Figure 2:**
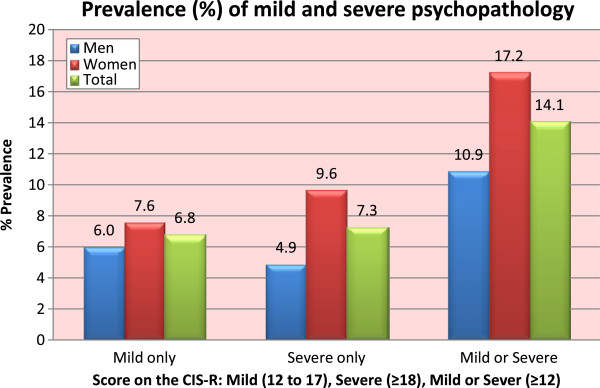
Prevalence of general psychopathology, N = 4894.

The CIS-R estimates the presence of 14 common psychiatric symptoms during the past week. Figure 1 presents the prevalence of symptoms by gender using a score of 2 or more as the cut-off for clinical significance (see methods). Fatigue, irritability and worry were the most common symptoms with a prevalence in the whole sample of >15%, while obsessions, phobias and panic were the least common with a prevalence of <5%. All symptoms were significantly more common in women (p < 0.001) except irritability (p = 0.26). Figure 2 shows the prevalence of general psychopathology by gender using the total CIS-R score to denote mild (score ≥12 & ≤17) or severe (score ≥18) psychopathology. In total, approximately 14.06% (95% CI: 13.08 – 15.03) of the population had either mild or severe psychopathology with a significant gender effect (p < 0.001, Figure 2). A detailed table of the distribution of the 14 symptoms assessed by the CIS-R in men, women and the total sample is given in the appendix (Additional file 1: Table S2).

### Prevalence of common mental disorders

The prevalence of common mental disorders by gender is shown in Table 1 (for a graphic representation by gender and age please see the Additional file 1: Figures S1 and S2 respectively). It can be seen that the most common disorder was generalized anxiety disorder (GAD) with a prevalence of 4.10% (95% CI: 3.54 – 4.65) followed by depressive episode at 2.90% (95% CI: 2.43 – 3.37). All psychiatric diagnoses were significantly more common in women except for the non-specific “mixed anxiety-depression” which was marginally non-significant (p = 0.08 for the latter; p < 0.001 for the remaining disorders except p = 0.03 for OCD). Criteria for at least one depressive or anxiety disorder were met by 7.67% of the population (95% CI: 6.92 – 8.41). Prevalence of depression showed an increase in the age groups 50–59 and 60–70, while that of anxiety disorders in the age group 60–70. Further analyses showed that this association was confounded by the presence of severe chronic medical conditions (see below on the sociodemographic associations section and in the Additional file 1: Figures S2, S2a and S2b).

**Table 1:** **Prevalence** (**1-week**) **of common mental disorders and substance use by gender in a representative sample of the general population of Greece 18–70, (weighted data, N = 4894)**

	**Male**	**Female**	**p-value**	**Total**
	**% (95% CI**^**1**^**)**	**% (95% CI**^**1**^**)**	**(male vs. female)**	**% (95% CI**^**1**^**)**
**Depressive Episode**	**2.10% (1.53 – 2.67)**	**3.68% (2.94 – 4.42)**	**0.001**	**2.90% (2.43 – 3.37)**
**Any Anxiety Disorder**	**4.49% (3.66 – 5.31)**	**8.45% (7.36 – 9.55)**	**<0.001**	**6.49% (5.80 – 7.18)**
Generalized Anxiety Disorder	2.55% (1.92 – 3.18)	5.62% (4.71 – 6.53)	**<0.001**	4.10% (3.54 – 4.65)
Panic Disorder	1.19% (0.76 – 1.62)	2.55% (1.93 – 3.17)	**<0.001**	1.88% (1.50 – 2.26)
Obsessive Compulsive Disorder	1.27% (0.83 – 1.72)	2.10% (1.54 – 2.67)	**0.025**	1.69% (1.33 – 2.05)
All Phobias	1.93% (1.39 – 2.43)	3.64% (2.90 – 4.38)	**<0.001**	2.79% (2.33 – 3.26)
**Any Anxiety or Depressive Disorder**	**5.43% (4.53 – 6.33)**	**9.87% (8.69 – 11.05)**	**<0.001**	**7.67% (6.92 – 8.41)**
**Other Non Specific Psychiatric Morbidity (“mixed anxiety-depression”)**	**2.26% (1.67 – 2.85)**	**3.07% (2.39 – 3.75)**	0.078	**2.67% (2.22 -3.12)**
**Harmful Alcohol Use**	**16.95% (15.45 – 18.44)**	**8.51% (7.40 – 9.60)**	**<0.001**	**12.69% (11.75 – 13.62)**
**Smoking Use (regular user - past month)**	**49.56% (47.57 – 51.56)**	**29.80% (28.0 – 31.61)**	**<0.001**	**39.60% (38.22 – 40.97)**
**Cannabis Use (at least once - past month)**	**3.54% (2.80 – 4.27)**	**0.61% (0.30 – 0.91)**	**<0.001**	**2.06% (1.66 – 2.46)**
**Score on the CIS-R ≥12**^**2**^	**10.84% (9.60 – 12.08)**	**17.21% (15.72 – 18.70)**	**<0.001**	**14.06% (13.08 – 15.03)**

^**1**^*CI* Confidence intervals, ^2^ A score on the CIS-R ≥12 represents a dimensional view of clinically significant psychiatric morbidity.

### Prevalence of substance use disorders

Harmful alcohol use, defined as a score of ≥6 for men and ≥4 for women in audit-c (see methods), was reported by 12.69% of the population (95% CI: 11.75 – 13.62) and was more common in men compared to women (16.95% vs. 8.51% respectively, p < 0.001) [Table 1]. Approximately 40% of the population reported regular smoking during the past month with a significant gender effect (50% for men vs. 30% for women, p < 0.001). The prevalence of Cannabis use during the past month was 2.06% (95% CI: 1.66 – 2.46) and it was also more common in men. Harmful alcohol use and smoking were less common in the age group 60–70 (p < 0.001 compared to 18–29), while cannabis use was higher in the age group 18–29 (p < 0.001 compared to all other age groups).

### Comorbidity, health status and health services use

**Figure 3:**
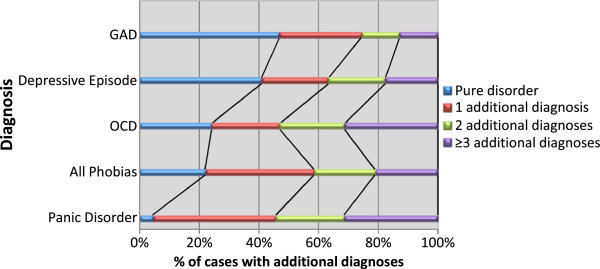
Comorbidity of common mental disorders.

Table 2 (left columns) shows comorbidity rates for five mental disorders. Panic disorder was rare in “pure” (i.e. non-comorbid) form, while depression and GAD were more common in pure forms (41% and 47% respectively). OCD and phobic disorders were in the middle with approximately one fifth of the cases being “pure”. Figure 3 shows a graphical representation of comorbidity rates.

**Table 2:** Co-morbidity, health status and use of mental health services of participants with common mental disorders in a representative sample of the general population of Greece (18–70), N = 4894

**Percentage (95% CI**^**1**^**) with**					**Health status (Score on the EQ-5D index) &**			
					**Use of services (% of cases who visited a Mental Health Professional past 12 months)**			
	**Pure disorder**	**1 additional diagnosis**	**2 additional diagnoses**	**≥3 additional diagnoses**		**Total**	**Pure disorder**	**≥1 additional diagnoses**
Depressive Episode	40.8%	22.5%	19.0%	17.6%	EQ-5D index (SD^2^)	0.46 (0.30)	0.46 (0.28)	0.46 (0.32)
	(33.7 – 49.0)	(15.6 – 29.5)	(12.5 – 25.5)	(11.3 – 23.9)	Visited MH Prof. ^3^	34.5%	27.6%	39.3%
GAD^4^	46.8%	27.4%	12.9%	12.9%	EQ-5D^2^ index (SD^2^)	0.51 (0.28)	0.53 (0.28)	0.49 (0.28)
	(39.8 -53.7)	(21.1 – 33.6)	(8.2 – 17.6)	(8.2 – 17.6)	Visited MH Prof. ^3^	29.3%	23.4%	34.6%
Panic Disorder	4.3%	41.3%	22.8%	31.5%	EQ-5D^2^ index (SD^2^)	0.49 (0.31)	0.22 (0.13)^7^	0.50 (0.31)^7^
	(0.1 – 8.6)	(31.0 – 51.6)	(14.1 – 31.6)	(21.8 – 41.2)	Visited MH Prof. ^3^	44.6%	50.0%	44.3%
OCD^5^	24.1%	22.9%	21.7%	31.3%	EQ-5D^2^ index (SD^2^)	0.55 (0.33)	0.68 (0.32)^8^	0.51 (0.32)^8^
	(14.7 – 33.5)	(13.7 – 32.1)	(12.6 – 30.7)	(21.1 – 41.5)	Visited MH Prof. ^3^	30.1%	10.0%^9^	36.5%^9^
All Phobias	21.9%	36.5%	20.4%	21.2%	EQ-5D^2^ index (SD^2^)	0.55 (0.31)	0.69 (0.26)^8^	0.51 (0.31)^8^
	(14.9 – 28.9)	(28.3 – 44.7)	(13.6 – 27.3)	(14.2 – 28.1)	Visited MH Prof. ^3^	40.9%	43.3%	40.2%
Score on the CIS-R ≥12^5^	**NA**^**6**^				EQ-5D^**2**^ index (SD^**2**^)	0.54 (0.30)	**NA**^**6**^	
					Visited MH Prof. ^**3**^	23.69%		
No current Psychiatric morbidity (score on the CIS-R <12)	**NA**^**6**^				EQ-5D^**2**^ index (SD^**2**^)	0.86 (0.18)	**NA**^**6**^	
					Visited MH Prof. ^**3**^	3.28%		

^**1**^*CI* Confidence intervals, ^**2**^*SD* Standard deviation, ^3^*MH Prof.* Mental health professional (either psychiatrist or psychologist), ^4^*GAD* Generalized anxiety disorder, ^5^*OCD* Obsessive compulsive disorder, ^6^ A score on the CIS-R ≥12 represents a dimensional view of clinically significant psychiatric morbidity; ^6^ NA: not applicable; ^7^p = 0.08 (EQ-5D - pure vs. comorbid); ^8^p < 0.05 (EQ-5D - pure vs. comorbid); ^9^p < 0.05 (Visited MH Prof - pure vs. comorbid).

Table 2 (right columns) also shows the health status (assessed with EQ-5D) and mental health service use (during the past 12 months) associated with the presence of common mental disorders in pure and comorbid forms. Health status was lower in comorbid forms of panic disorder, OCD and phobic disorders, while there were no significant differences for depression and GAD. Compared to participants without psychiatric morbidity, scores on the health status index were significantly lower for those with psychiatric morbidity (0.52 [sd: 0.30] vs 0.84 [sd: 0.20] for all common mental disorders combined, p < 0.001). Regarding mental health service use, roughly one third (~32%) of those with a current common mental disorder reported that they had visited a mental health professional during the past year. Service use was more common in panic disorder (approximately 45% of those with current panic had seen a mental health professional in the past 12 months). With the exception of panic, participants with comorbid forms of disorders had visited more often a mental health professional but this reached statistical significance only for OCD where only 10% with “pure” form had visited a professional compared to 36.5% of the comorbid form (p = 0.024).

### Sociodemographic associations

Table 3 presents adjusted odds ratios and their 95% confidence intervals for the association between sociodemographic variables and common mental disorders/harmful alcohol use (for crude odds ratios please see Additional file 1: Table S3). We also present the associations for our dimensional definition of psychiatric morbidity (CIS-R ≥12) for comparison. Anxiety disorders have been grouped into one category (data for specific disorders are available upon request by authors).

**Table 3:** Sociodemographic associations of common mental disorders and harmful alcohol use in a representative sample of the general population of Greece (18–70), N = 4894

	**Depressive episode**			**Any anxiety disorder**			**Harmful alcohol use**			**Score on the CIS-R ≥12**^**1**^		
	**%**	**Adjusted OR**^**2**^	**95% CI**^**3**^	**%**	**Adjusted OR**^**2**^	**95% CI**^**3**^	**%**	**Adjusted OR**^**2**^	**95% CI**^**3**^	**%**	**Adjusted OR**^**2**^	**95% CI**^**3**^
**Gender**												
Men	2.10%	1.00	Ref	4.49%	1.00	Ref	16.95%	1.00	Ref	10.85%	1.00	Ref
Women	3.68%	**1.66**	1.12 – 2.47	8.45%	**2.12**	**1.61 – 2.78**	8.51%	**0.53**	**0.43 – 0.64**	17.21%	**1.69**	**1.39 – 2.05**
**Age**												
18-29	1.38%	1.00	Ref	4.64%	1.00	Ref	14.85%	1.00	Ref	8.16%	1.00	Ref
30-39	2.03%	1.58	0.75 – 3.34	5.80%	1.19	0.76 – 1.87	13.86%	0.99	0.75 – 1.31	10.95%	1.29	0.92 – 1.80
40-49	2.46%	1.65	0.74 – 3.65	6.52%	1.16	0.72 – 1.88	13.70%	1.00	0.73 – 1.37	14.78%	**1.58**	**1.11 – 2.24**
50-59	3.86%	2.11	0.94 – 4.73	6.84%	0.97	0.58 – 1.63	12.97%	0.87	0.61 – 1.24	16.83%	1.41	0.97 – 2.05
60-70	5.56%	2.05	0.85 – 4.94	9.44%	0.92	0.51 – 1.65	7.11%	**0.40**	**0.25 – 0.65**	22.44%	1.29	0.84 – 1.96
**Marital Status**												
Married	2.74%	1.00	Ref	6.24%	1.00	Ref	11.15%	1.00	Ref	14.76%	1.00	Ref
Single	1.52%	1.11	0.57 – 2.13	4.08%	0.79	0.52 – 1.21	16.11%	**1.41**	**1.09 – 1.84**	8.23%	0.88	0.65 – 1.19
Divorced/Separated	5.83%	**2.10**	**1.13 – 3.90**	12.08%	**1.89**	**1.22 – 2.93**	18.33%	**1.77**	**1.23 - 2.54**	22.50%	**1.67**	**1.19 – 2.34**
Widowed	11.27%	**2.44**	**1.39 – 4.30**	16.43%	**1.66**	**1.05 – 2.63**	4.69%	0.57	0.29 – 1.13	34.27%	**1.72**	**1.21 – 2.44**
**Educational Qual.**												
None/Primary Educ.	5.40%	1.00	Ref	9.72%	1.00	Ref	12.63%	1.00	Ref	23.65%	1.00	Ref
Lower Secondary	4.27%	1.18	0.72 – 1.93	7.28%	0.99	0.68 – 1.45	15.18%	0.79	0.59 – 1.07	19.32%	1.05	0.81 – 1.36
Upper Secondary	1.92%	0.67	0.40 – 1.14	5.15%	0.81	0.55 – 1.17	12.65%	**0.55**	**0.41 – 0.73**	10.14%	**0.60**	**0.46 – 0.78**
Technical Vocational	1.82%	0.61	0.26 – 1.42	4.56%	0.69	0.39 – 1.22	10.02%	**0.41**	**0.27 – 0.62**	8.66%	**0.49**	**0.33 – 0.74**
Tertiary Education	1.30%	**0.40**	**0.15 – 1.09**	5.47%	0.82	0.47 – 1.44	10.94%	**0.50**	**0.32 – 0.75**	10.16%	**0.56**	**0.37 – 0.84**
**Employment Status**												
Full-time/part-time	2.19%	1.00	Ref	5.42%	1.00	Ref	14.91%	1.00	Ref	11.62%	1.00	Ref
Looking after house	3.47%	0.73	0.41 – 1.30	6.80%	**0.64**	**0.43 – 0.95**	6.37%	**0.60**	**0.42 – 0.87**	17.95%	0.80	0.60 – 1.06
Unemployed	5.43%	**2.46**	**1.20 – 5.04**	9.24%	1.58	0.92 – 2.73	19.57%	1.37	0.93 – 2.04	18.48%	**1.68**	**1.12 – 2.54**
Retired	5.37%	0.72	0.39 – 1.31	9.88%	0.88	0.56 – 1.39	8.32%	0.89	0.58 – 1.37	22.36%	0.85	0.61 – 1.17
Other Econ. Inactive	2.49%	1.04	0.54 – 1.99	5.93%	1.06	0.69 – 1.64	11.09%	**0.69**	**0.50 – 0.94**	11.85%	1.06	0.77 – 1.47
**Type of locality**												
Urban	3.02%	1.00	Ref	6.07%	1.00	Ref	10.07%	1.00	Ref	13.50%	1.00	Ref
Semi-rural	1.97%	0.58	0.31 – 1.08	7.39%	1.18	0.82 – 1.69	15.16%	**1.53**	**1.18 - 1.99**	14.66%	1.02	0.78 – 1.33
Rural	3.05%	0.93	0.64 – 1.36	6.85%	1.13	0.87 – 1.48	16.14%	**1.61**	**1.33 – 1.94**	14.77%	1.01	0.84 – 1.23
**Presence of Chronic Physical Diseases**												
No	1.01%	1.00	Ref	2.49%	1.00	Ref	12.92%	1.00	Ref	5.70%	1.00	Ref
Yes	5.80%	**3.5**	**2.34 – 5.24**	12.64%	**3.96**	**2.96 – 5.31**	12.33%	1.22	0.92 – 1.62	26.89%	**3.62**	**2.92 – 4.50**

^1^ A score on the CIS-R ≥12 represents a dimensional view of clinically significant psychiatric morbidity; ^**2**^*OR* Odds Ratio adjusted for all other variables of the table; ^**3**^*CI* Confidence intervals. Figures in bold statistically significant at p < 0.05.

For depression, female gender, being divorced/separated or widowed and being unemployed were associated with an increased prevalence, while having a better education with a lower prevalence (p = 0.024 for the linear trend in education). For anxiety disorders, female gender and being divorced/separated or widowed was associated with an increased prevalence. Harmful alcohol consumption was less likely in women, the older age group, the better educated (p < 0.001 for the linear trend in education) and for those looking after their house or were economically inactive. It was higher in single persons and the divorced/separated. Presence of a chronic severe medical condition was also strongly associated with both depression or anxiety disorders but not with harmful alcohol use (Table 3).

### Duration of common mental disorders

**Figure 4:**
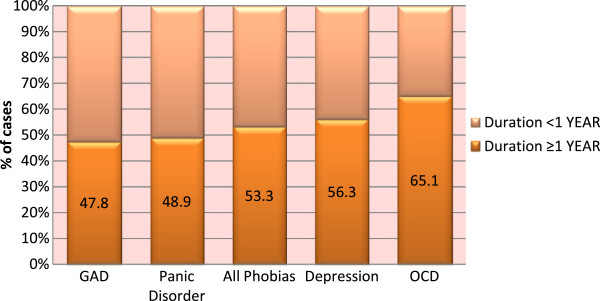
Duration of common mental disorders.

We retrospectively assessed the duration of the core symptoms of common mental disorders and in Figure 4 we present the percentage of cases with a duration of symptoms for at least one year (or more). It can be seen that chronicity (duration of illness for one year or more) was reported by approximately half of those with a common mental disorder. OCD and depression had the longest duration with 65% and 56% persistence over one year respectively (Figure 4).

## Discussion

### Main findings

In this nationally representative sample of the Greek general population the one-week prevalence of general psychiatric morbidity of at least mild severity was 14.06% (95% CI: 13.08 – 15.03). Generalized anxiety disorder and depressive episode were the most common specific disorders. A pattern of harmful alcohol use was noted in 12.69% (11.75 – 13.62) of the population. Women were more likely to report a common mental disorder but less likely to report use or abuse of all substances studied. All disorders were associated with a considerable reduction in health related quality of life and a duration of symptoms longer than one year was common. Despite these characteristics, only one third of those with a disorder reported use of mental health services within the past 12 months.

### Limitations

These findings should be interpreted in the context of the following limitations: a) The response rate of the survey was relatively low at 54.2%. This is typical of what can be achieved in general population surveys in countries without considerable tradition in medical or psychiatric survey research, such as Greece. The possibility of selection bias cannot be ruled out, although the age and sex distribution of the participants was comparable to the national data. In any case, we have weighted our results to account for non-response; b) The “clinical” validity of the common mental disorders we have defined in this paper has not been studied in the context of the present study. A few studies in the past have tried to test the validity of psychiatric diagnoses obtained by structured diagnostic instruments administered by non-clinicians, and generally report low to moderate concordance with all diagnostic interviews [25-27]; c) Chronic physical illnesses were assessed crudely by self-report and this will be inaccurate. In addition, the duration of psychiatric symptoms was retrospectively assessed and there is a risk for recall bias which could lead to overestimation depending on the severity of the current episode of illness; d) Finally, as this is a cross-sectional study, any reported associations between the common mental disorders and other variables do not have any causal implications. Reverse causality may be equally true for some of the reported associations.

### Comparison with previous studies in Greece

We have previously carried out a number of smaller scale studies in Greece using the CIS-R to assess psychiatric morbidity. Two of them were carried out in islands of the Aegean sea, the first in small Northern Aegean communities [28,29] and the second in Paros [30]. In all studies, fatigue irritability and worry were the three more prevalent symptoms, although in the small island communities general psychiatric morbidity was reported higher, especially in Paros where 30% of the women and 13% of men were found to have a CIS-R score of ≥12 compared to 17% and 11% respectively in the current study. These differences are more likely explained by the sampling procedure that resulted in an overrepresentation of women in the Paros study. Differences in the Northern Aegean study were smaller. A third study was carried out in older adolescents (16–18 years old) attending senior high schools in Greece and since this was carried out in the school context and in this specific age group results are more difficult to compare [17].

There are two previous general population surveys of psychiatric morbidity in Greece, both carried out by Madianos and his colleagues in 1978 and 1984 [7]. Samples (of 4083 and 3706 participants respectively) were drawn from mainland Greece only, excluding households from insular Greece. Psychiatric morbidity was assessed with Langner’s scale (an instrument which predated modern diagnostic criteria) and the Center for Epidemiologic Studies of Depression Scale (CES-D). Due to their design, these studies have provided data on general psychiatric morbidity. Regarding specific diagnostic categories only major depression was covered. These studies reported clinical significant psychiatric morbidity in 24% of women and 11% of men in 1978 and in 34% vs 19% respectively in 1984. Prevalence of depression was reported by 4.5% of women and 2.4% of men in 1978 and 6.6% vs 3.8% respectively in 1984 [7]. These figures and especially those from the 1984 study are considerably higher than the ones reported in the present study, but the different instruments and sampling frame are probably the major source of discrepancy. During the same period, Mavreas et al. [31] carried out a smaller scale study in Athens (N = 489) using a structured psychiatric interview, the Present State Examination (PSE) to assess psychiatric disorders. It is worth noting that in that study general psychiatric morbidity was reported by 16% of the population, very similar to the one reported here, given the differences in methods and the length of time interval (24 years) between the two studies. More recently, a number of telephone cross-sectional surveys were organized in Greece with the aim to assess depression [8,9]. Three such studies have documented an increasing trend in the prevalence of depression (assessed with the depression module of the Structured Clinical Interview – SCID-I), from 3.3% in 2008, to 6.8% in 2009 and 8.2% in 2011. The present study, carried out in 2009–2010, has reported a lower figure for the prevalence of depression than the ones reported in these telephone surveys. It would be interesting to investigate whether this is due to the application of diagnostic criteria or there is a higher reporting of the core depressive symptoms in the telephone surveys compared to the structured clinical interview we used in the present study. It is likely that the use of structured psychiatric interviews yields more conservative estimates of clinically significant disorders, while the methods used in the telephone surveys are more likely to result in over-reporting of milder forms of psychopathology especially in those above 60 [32]. It is worth noting that our estimates of depression and anxiety disorders are associated with low health-related quality of life (Table 2) and substantial chronicity, arguing in favor of a higher clinical significance threshold in our study compared to these telephone surveys. A recent study in four European sites has also confirmed that the CIS-R is less sensitive but more specific in its depression diagnosis compared to other structured interviews such as the composite international diagnostic interview [15].

Regarding alcohol and other substances our results are compatible with previous surveys in Greece that show high rates for smoking and low for cannabis compared to other European countries [33-35]. Most previous studies of alcohol in Greece have taken a more clinical approach and therefore it is difficult to compare their results with our study where we have used a more dimensional approach. It should be noted however that all associations with harmful alcohol use were in the expected direction supporting the validity of our approach.

### Comparison with other studies in Europe and elsewhere

Wittchen & Jacobi in 2005 [36] reviewed 27 general adult population studies conducted in Europe and have recently (2011) updated this review extending their coverage to children and adolescent studies [5]. It should be noted that our results refer to 1-week prevalence while the review used data on 12-month prevalence. Based on previous studies (see for example [37] and [38]) that have reported both one-month and 12-month prevalence of common mental disorders, a conservative estimate for the ratio of one year vs. one month prevalence is 1.5 for depression and 1.3 for anxiety disorders which translates to projected estimates of 12-month prevalence for our study of 4.3% for depression and 8.4% for anxiety disorders. Comparing these estimates to the other European studies one can see that depression is on the lower side (median prevalence in European studies of 5.7% vs 4.3% projected in the present) while generalized anxiety (median 2% vs. 5.3% projected) and other anxiety disorders are on the higher (with the exception of phobias).

Another interesting study that can be used for comparison is the World Mental Health Survey that was carried out in 14 countries from all continents [6]. According to this study, our prevalence estimates would place Greece in the middle regarding prevalence of depression along with Germany, Italy, Mexico and Spain (with Asian countries in the low and US and other Northern European countries such as France, Netherlands and Ukraine in the high). Regarding anxiety disorders, this would be middle to high. A similar pattern of lower prevalence of depression compared to anxiety disorders and especially generalized anxiety disorder had also been reported from Athens in a WHO cross-cultural study of common mental disorders in primary care [39]. It is also reminded that rates of suicide, often associated with severe depression, in Greece are among the lowest in the world [40]. It is possible that socio-cultural factors, including strong family cohesion, high levels of social support and higher religiosity, may play an important role in explaining these differences.

### Sociodemographic associations

Most of the associations reported for common mental disorders (e.g. female gender, marital status, educational status) are in the expected direction and confirm the findings of previous studies both in Greece and elsewhere. These have been discussed thoroughly in previous reviews [5]. We would like to comment here on the association between common mental disorders and age. In the unadjusted analysis there was a significant association between older age and depression or anxiety disorders. The effect was stronger for depression (for example prevalence of depression in the older age group was 5.56% vs 1.38% in the younger group, p < 0.001, while for anxiety disorders was 9.44% vs 4.64% respectively, p < 0.001). This same finding has been also reported by other surveys in Greece for depression (see for example [8]) or for general psychiatric morbidity assessed in health-related quality of life instruments (see for example validation studies of EQ-5D [23] and SF-36 [41]. Traditionally, in studies carried out in developed countries, older age is usually associated with a reduced prevalence [11,42,43]. It is worth noting that in our adjusted analysis the age effect was greatly reduced and became non-significant. This was mainly due to the inclusion into the model of the presence of a comorbid chronic medical condition. There is a strong association between chronic physical disorders and common mental disorders [44,45] and this was also confirmed in our study. As the prevalence of chronic medical conditions increase sharply with age, this creates a spurious association between older age and depression or anxiety that is due to the confounding effect of the comorbid chronic physical disorders. It is difficult however to explain why this is shown in the Greek studies and not in other epidemiological surveys elsewhere, where the association between chronic physical disorders and mental disorders is also strong [45]. One possibility is the less optimal management of chronic physical disorders in the Greek health system which is mostly focused on the medical dimension and not on the overall quality of life including mental health issues. The Greek health system is still physician-centred rather than multidisciplinary [46]. This issue merits further investigation in the future as the older age group appears quite affected by both mental and physical disorders and their combined harmful effect in quality of life has not been recognized and appreciated in Greece.

Unemployment was also associated with common mental disorders and especially with depression, a common finding in epidemiological research [47]. However, in Greece there has been a sharp increase in unemployment recently and the health effects of unemployment will become a serious public health issue, taking into account the difficulty in establishing an effective safety net for the unemployed in an economy which is in deep recession [48]. In a companion paper we examine more in detail the association between various socioeconomic indicators and unemployment and common mental disorders and suicidal behaviour (Skapinakis et al. in preparation).

### Use of services

Use of mental health services during the past 12 months was reported by approximately one third of those who met criteria for a psychiatric diagnosis, a common finding in several surveys around the world [49]. It was higher for panic disorder and for comorbid vs. pure disorders with the exception of panic and phobic anxiety. An analysis of the correlates of service use is beyond the scope of the current paper and it will be explored in more detail in future reports.

## Conclusions

We presented the results of the first general population survey of psychiatric morbidity that used a nationally representative sample from both mainland and insular Greece. These results show that a considerable proportion of the population suffer from common mental disorders. In this figure we should also add those suffering from serious mental disorders not covered in the present survey, such as bipolar disorder or schizophrenia. These results highlight the need for better organization of services taking into account that the majority of those with a mental illness will not visit a mental health professional either due to stigmatization [50] or low recognition [39]. This is even more important today in Greece because of the restrictions imposed on the health budget that could disproportionately affect funds available for mental health. Our study also highlights the close association between mental and physical disorders supporting the view that there is no health without mental health [51]. Our finding that depression and anxiety are greatly increased in older people with chronic physical disorders also calls for a more careful assessment of common mental disorders in this group. In times of economic crises, the observed increases in morbidity and mortality is the result of common diseases that are less optimally managed. The Russian example during the 90’s is quite informative: the mortality fluctuations observed there during the 90’s were mainly due to changes in mortality from vascular diseases and harmful alcohol use [52]. The additional burden of mental illness in this group of patients may also be a contributing factor that needs to be taken into account if we want to reduce the impact of the economic crisis in the health of the population.

## Competing interests

The authors declare that they have no competing interests.

## Authors’ contributions

PS was responsible (with VM) for the conception and design of the study, helped in data collection, contributed to the statistical analysis and drafted the manuscript. SB helped in data collection, in the statistical analysis, in the writing of the manuscript and interpretation of the results. SK helped in obtaining funding for the study and contributed to the writing of the paper and interpretation of the results. IG helped in data collection, writing of the manuscript and interpretation of the results. PNT helped in data collection, writing of the manuscript and interpretation of the results. VM was responsible (with PS) for the conception and design of the study and helped in obtaining funding for the study, in the writing of the paper and interpretation of the results. All authors read and approved the final manuscript.

## Pre-publication history

The pre-publication history for this paper can be accessed here:

http://www.biomedcentral.com/1471-244X/13/163/prepub

## Supplementary Material

Additional file 1: Table S1 Basic Description of the Sample in a representative sample of the general population of Greece (18-70, N = 4894). **Table S2.** Prevalence of Psychiatric Symptoms by gender in a representative sample of the general population of Greece (18-70, N = 4894). **Table S3.** Crude odds ratios of the association between sociodemographic associations and common mental disorders/harmful alcohol use in a representative sample of the general population of Greece (18-70, N = 4894). **Figure S1.** Prevalence of common mental disorders by gender in a representative sample of the general population of Greece (18-70), N = 4894. **Figure S2.** Age distribution of common mental disorders in a representative sample of the general population of Greece (18-70), N = 4894. Click here for file
