# Supplementary material for: Prevalence and sociodemographic associations of common mental disorders in a nationally representative sample of the general population of Greece
Source: BMC Psychiatry. 2013 Jun 4;13:163. doi: 10.1186/1471-244X-13-163 (PMC3686601; doi:10.1186/1471-244X-13-163)
Supplement: Additional file 1: Table S1 — Basic Description of the Sample in a representative sample of the general population of Greece (18-70, N = 4894). Table S2. Prevalence of Psychiatric Symptoms by gender in a representative sample of the general population of Greece (18-70, N = 4894). Table S3. Crude odds ratios of the association between sociodemographic associations and common mental disorders/harmful alcohol use in a representative sample of the general population of Greece (18-70, N = 4894). Figure S1. Prevalence of common mental disorders by gender in a representative sample of the general population of Greece (18-70), N = 4894. Figure S2. Age distribution of common mental disorders in a representative sample of the general population of Greece (18-70), N = 4894. [file 1471-244X-13-163-S1.doc]

# Additional file

Prevalence and sociodemographic associations of common mental disorders in a nationally representative sample of the general population of Greece

Petros Skapinakis et al.

# *Additional file 1: Tables, S1, S2 & S3*

# *Additional file 1: Figures, S1, S2, S2a & S2b*

# *Additional file (Supplemental Material not for review)*

**Additional file 1: Table S1. Basic Description of the Sample in a representative sample of the general population of Greece (18-70, N=4894).**

|  | **N** | **%** |
| --- | --- | --- |
| **Gender**  Men  Women | 2425  2469 | 49.55 %  50.45 % |
| **Age**  18-29  30-39  40-49  50-59  60-70 | 1226  1032  934  802  900 | 25.05 %  21.09 %  19.08 %  16.39 %  18.39 % |
| **Marital Status**  Married  Single  Divorced / Separated  Widowed | 2995  1,446  240  213 | 61.20 %  29.55 %  4.90 %  4.35 % |
| **Educational Qual.**  None / Primary Education  Lower Secondary Education  Upper Secondary Education  Technical Vocational Education  Tertiary Education | 926  797  2348  439  384 | 18.92 %  16.28 %  47.98 %  8.97 %  7.85 % |
| **Employment Status**  Full-time / part-time  Looking after house  Unemployed  Retired  Other Economically Inactive | 2917  691  184  577  525 | 59.60 %  14.12 %  3.76 %  11.79 %  10.73 % |
| **Type of locality**  Urban  Semi-rural  Rural | 2682  607  1605 | 54.80 %  12.40 %  32.80 % |
| **Presence of Chronic Physical Diseases**  No  Yes | 4235  659 | 86.53 %  13.47 % |

**Additional file 1: Table S2. Prevalence of Psychiatric Symptoms by gender in a representative sample of the general population of Greece (18-70, N=4894).**

| **Symptom** | **Prevalence % of clinically significant symptoms**  **(95% CI^1^)** | **p-value^2^** |
| --- | --- | --- |
| **Fatigue**  Male  Female  Total | 18.93% (17.37 – 20.49)  28.31% (26.53 – 30.09)  23.66% (22.47 – 24.85) | p<0.001 |
| **Irritability**  Male  Female  Total | 18.93% (17.37 – 20.49)  20.21% (18.63 – 21.80)  19.57% (18.46 – 20.69) | p=0.26 |
| **Worry**  Male  Female  Total | 15.46% (14.02 – 16.90)  21.30% (19.69 – .22.92)  18.41% (17.32 – 19.50) | p<0.001 |
| **Depressive Mood**  Male  Female  Total | 10.97% (9.72 – 12.21)  15.67% (14.24 – 17.11)  13.34% (12.39 – 14.30) | p<0.001 |
| **Sleep Problems**  Male  Female  Total | 9.65% (8.47 – 10.83)  14.05% (12.68 – 15.43)  11.87% (10.97 – 12.78) | p<0.001 |
| **Psychosomatic Symptoms**  Male  Female  Total | 7.38% (6.34 – 8.42)  15.15% (13.73 – 16.56)  11.30% (10.41 – 12.19) | p<0.001 |
| **Depressive Ideas**  Male  Female  Total | 8.82% (7.69 – 9.95)  13.73% (12.37 – 15.09)  11.30% (10.41 – 12.19) | p<0.001 |
| **Anxiety (free-floating)**  Male  Female  Total | 7.59% (6.53 – 8.64)  11.83% (10.55 – 13.10)  9.73% (8.90 – 10.56) | p<0.001 |
| **Worry about physical health**  Male  Female  Total | 5.48% (4.58 – 6.39)  7.94% (6.87 – 9.01)  6.72% (6.02 – 7.42) | p<0.001 |
| **Compulsions**  Male  Female  Total | 3.75% (3.00 – 4.51)  7.13% (6.11 – 8.14)  5.46% (4.82 – 6.09) | p<0.001 |
| **Concentration / Memory Problems**  Male  Female  Total | 3.46% (2.74 – 4.19)  6.76% (5.77 – 7.76)  5.13% (4.51 – 5.75) | p<0.001 |
| **Obsessions**  Male  Female  Total | 3.55% (2.81 – 4.28)  5.22% (4.35 – 6.10)  4.39% (3.82 – 4.97) | p=0.004 |
| **Phobias**  Male  Female  Total | 2.93% (2.26 – 3.60)  5.22% (4.35 – 6.10)  4.09% (3.53 – 4.64) | p<0.001 |
| **Panic**  Male  Female  Total | 1.44% (0.97 – 1.92)  3.40% (2.69 – 4.12)  2.43% (2.00 – 2.86) | p<0.001 |

^1^ CI: confidence intervals; ^2^ p-values for the gender difference.

**Additional file 1: Table S3 Crude odds ratios of the association between sociodemographic associations and common mental disorders / harmful alcohol use in a representative sample of the general population of Greece (18-70, N=4894).**

|  | *Depressive Episode* | | *Any Anxiety Disorder* | | *Harmful Alcohol Use* | | **Score on the CIS-R ≥12^1^** | |
| --- | --- | --- | --- | --- | --- | --- | --- | --- |
|  | **Crude OR^2^** | **95% CI^3^** | **Crude OR^2^** | **95% CI^3^** | **Crude OR^2^** | **95% CI^3^** | **Crude OR^2^** | **95% CI^3^** |
| **Gender**  Men  Women | 1.00  **1.78** | Ref  1.26 – 2.52 | 1.00  **1.97** | Ref  **1.55 – 2.50** | 1.00  **0.46** | Ref  **0.38 – 0.54** | 1.00  **1.71** | Ref  **1.45 – 2.02** |
| **Age**  18-29  30-39  40-49  50-59  60-70 | 1.00  1.47  1.80  **2.86**  **4.19** | Ref  0.78 – 2.81  0.96 – 3.39  **1.57 – 5.20**  **2.40 – 7.32** | 1.00  1.27  1.44  **1.51**  **2.14** | Ref  0.87 – 1.84  0.99 -2.08  **1.03 – 2.21**  **1.52 – 3.03** | 1.00  0.92  0.91  0.85  **0.44** | Ref  0.73 – 1.17  0.71 – 1.16  0.66 – 1.11  **0.33 – 0.59** | 1.00  **1.38**  **1.95**  **2.28**  **3.26** | Ref  **1.04 – 1.84**  **1.49 – 2.56**  **1.73 – 3.00**  **2.52 – 4.22** |
| **Marital Status**  Married  Single  Divorced / Separated  Widowed | 1.00  **0.55**  **2.20**  **4.51** | Ref  **0.34 – 0.88**  **1.22 – 3.94**  **2.80 – 7.28** | 1.00  **0.64**  **2.06**  **2.95** | Ref  **0.47 – 0.86**  **1.36 – 3.13**  **2.00 – 4.37** | 1.00  **1.53**  **1.79**  **0.39** | Ref  **1.28 – 1.83**  **1.27 –-2.53**  **0.21 – 0.75** | 1.00  **0.52**  **1.68**  **3.01** | Ref  **0.42 – 0.64**  **1.22 – 2.31**  **2.23 – 4.07** |
| **Educational Qual.**  None / Primary Educ.  Lower Secondary  Upper Secondary  Technical Vocational  Tertiary Education | 1.00  0.78  **0.34**  **0.33**  **0.23** | Ref  0.49 -1.22  **0.23 – 0.52**  **0.15 – 0.69**  **0.09 – 0.58** | 1.00  **0.73**  **0.51**  **0.44**  **0.54** | Ref  **0.52 – 1.03**  **0.38 – 0.67**  **0.27 – 0.73**  **0.33 – 0.88** | 1.00  1.23  1.00  0.77  0.85 | Ref  0.94 – 1.63  0.80 – 1.26  0.53 – 1.11  0.58 – 1.23 | 1.00  **0.77**  **0.36**  **0.31**  **0.37** | Ref  **0.61 – 0.98**  **0.30 – 0.46**  **0.21 – 0.44**  **0.25 – 0.53** |
| **Employment Status**  Full-time / part-time  Looking after house  Unemployed  Retired  Other Econ. Inactive | 1.00  1.60  **2.56**  **2.53**  1.13 | Ref  0.99 -2.58  **1.29 – 5.08**  **1.63 – 3.92**  0.62 – 2.08 | 1.00  1.27  **1.78**  **1.91**  1.10 | Ref  0.91 – 1.78  **1.05 – 3.00**  **1.39 – 2.63**  0.74 – 1.64 | 1.00  **0.39**  1.39  **0.52**  **0.71** | Ref  **0.28 – 0.53**  **0.95 – 2.03**  **0.38 – 0.71**  **0.53 – 0.95** | 1.00  **1.66**  **1.72**  **2.19**  1.02 | Ref  **1.33 – 2.08**  **1.17 – 2.54**  **1.75 – 2.75**  0.77 – 1.36 |
| **Type of locality**  Urban  Semi-rural  Rural | 1.00  0.65  1.01 | Ref  0.35 – 1.19  0.71 – 1.45 | 1.00  1.23  1.14 | Ref  0.88 – 1.74  0.89 – 1.46 | 1.00  **1.60**  **1.72** | Ref  **1.24 – 2.06**  **1.43 – 2.06** | 1.00  1.10  1.11 | Ref  0.86 – 1.41  0.93 – 1.33 |
| **Presence of Chronic Physical Diseases**  No  Yes | 1.00  **4.62** | Ref  **3.27 – 6.53** | 1.00  **4.04** | Ref  **3.15 – 5.18** | 1.00  0.91 | Ref  0.71 – 1.18 | 1.00  **4.38** | Ref  **3.63 – 5.28** |

^1^A score on the CIS-R ≥12 represents a dimensional view of clinically significant psychiatric morbidity; ^2^ OR: Odds Ratio; ^3^ CI: confidence intervals

Additional file

## Additional file 1: Figure S1 - Prevalence of common mental disorders by gender in a representative sample of the general population of Greece (18-70), N=4894.

## Additional file 1: Figure S2 - Age distribution of common mental disorders in a representative sample of the general population of Greece (18-70), N=4894.

**S2a. Prevalence in those with chronic medical disorders**

**S2b. Prevalence in those without**

**r***

**ic medical disorders**
